# Functional display of heterotetrameric human protein kinase CK2 on *Escherichia coli*: a novel tool for drug discovery

**DOI:** 10.1186/s12934-015-0263-z

**Published:** 2015-06-03

**Authors:** Andreas Gratz, Andre Bollacke, Sara Stephan, Christian Nienberg, Marc Le Borgne, Claudia Götz, Joachim Jose

**Affiliations:** Institut für Pharmazeutische und Medizinische Chemie, PharmaCampus, Westfälische Wilhelms-Universität Münster, Corrensstr. 48, 48149 Münster, Germany; Lehrstuhl für Biophysik, Ruhr-Universität Bochum, Universitätsstr. 150, 44801 Bochum, Germany; Université de Lyon, Université Lyon 1, Faculté de Pharmacie-ISPB, EA 4446 Biomolécules Cancer et Chimiorésistances, SFR Santé Lyon-Est CNRS UMS3453-INSERM US7, 69373 Lyon Cedex 8, France; Medizinische Biochemie und Molekularbiologie, Universität des Saarlandes, Kirrberger Str., Geb. 44, 66421 Homburg, Germany

**Keywords:** Assay, Autodisplay, CK2, Heterotetramer, Protein kinase, Screening, Surface display, Drug discovery

## Abstract

**Background:**

Human protein kinase CK2 represents a novel therapeutic target for neoplastic diseases. Inhibitors are in need to explore the druggability and the therapeutic options of this enzyme. A bottleneck in the search for new inhibitors is the availability of the target for testing. Therefore an assay was developed to provide easy access to CK2 for discovery of novel inhibitors.

**Results:**

Autodisplay was used to present human CK2 on the surface of *Escherichia coli*. Heterotetrameric CK2 consists of two subunits, α and β, which were displayed individually on the surface. Co-display of CK2α and CK2β on the cell surface led to the formation of functional holoenzyme, as demonstrated by NaCl dependency of enzymatic activity, which differs from that of the catalytic subunit CK2α without β. In addition interaction of CK2α and CK2β at the cell surface was confirmed by co-immunoprecipitation assays. Surface displayed CK2 holoenzyme enabled an easy IC_50_ value determination. The IC_50_ values for the known CK2 inhibitors TBB and Silmitasertib were determined to be 50 and 3.3 nM, respectively.

**Conclusion:**

Surface-displayed CK2α and CK2β assembled on the cell surface of *E. coli* to an active tetrameric holoenzyme. The whole-cell CK2 autodisplay assay as developed is suitable for inhibition studies. Furthermore, it can be used to determine quantitative CK2 inhibition data such as IC_50_ values. In summary, this is the first report on the functional surface display of a heterotetrameric enzyme on *E. coli*.

## Background

To display a functional protein on the surface of a living cell bears several advantages in comparison to intracellular expression [1]. The protein at the cell surface is freely accessible for any kind of binding or activity studies. This can be applied e.g. in assays that allow to quantify protein–protein interactions by displaying one partner protein at the cell surface and subsequently add the binding partner protein labeled with a fluorescent dye, and determine the interaction by quantifying the whole cell fluorescence obtained thereby with the help of flow cytometry [2]. The screening of antibody libraries can be performed in a similar way, when the variants are displayed on the cell surface. The labeled antigen can be used, binding can be quantified by flow cytometry, and finally single cells bearing a distinct variant can be selected by fluorescence activated cell sorting [3, 4]. Such strategy can also be applied to identify novel enzyme inhibitors, taking into account that inhibitors have a high affinity (or a low dissociation constant) for enzymes they are inhibiting. In such case, peptide libraries displayed on the surface of *E. coli* can be screened by fluorescence labeled target enzymes, and whole cell fluorescence can be used for selecting strong binding variants [5, 6]. At this point it needs to be emphasized, that the libraries actually consist of different cells of *E. coli*, each displaying a different variant. Moreover one needs to have in mind that binding does not necessarily mean inhibition of the enzyme, but subsequently the selected variants can be tested on enzyme inhibition, and proofed to be a reliable source for novel inhibitors. Beside these activa for screening purposes, the cellular surface display of a protein, namely an enzyme has also advantages in the development of whole cell biocatalysts [7]. Neither the substrate nor the product need to cross a membrane barrier, and the formation of by-products by other enzymes is prevented, as it could appear by whole cell biocatalysts with intracellular enzyme expression. This is in particular an issue in the biocatalytic production of pharmaceuticals. Moreover, being anchored at the cell surface increases enzymatic stability [8], and in case the enzyme needs a membrane environment to be active this can be provided as well [9, 10]. A disadvantage of whole cell biocatalysts with surface displayed enzymes is, however, that enzymes which need supply of co-factors, e.g. redox co-factors like NADH or NAD^+^, are excluded from the regeneration circles, as available in the cytoplasm. For many of the applications mentioned here, autodisplay has been proven to be a helpful tool. In principle, autodisplay is the surface display of a recombinant protein in a Gram-negative bacterium by means of the autotransporter secretion pathway [11–13]. Nevertheless, most of the applications described until now are for the AIDA-I autotransporter to display a recombinant protein on *E. coli* [1]. One advantage of using the AIDA-I autotransporter for surface display in *E. coli* is the high number of displayed proteins, which were reported to be in the range of 10^5^–10^6^ [14, 15]. A second advantage is, that the anchoring domain, the so-called β-barrel, is not covalently linked to the surface, but can move or “swim” within the plane of the outer membrane. This can lead to a passenger driven dimerization or even multimerization at the cell surface, by simple expression and surface display of the corresponding monomer [16]. More recently it has been sown, that the co-display of a lipase and its specific foldase by AIDA-I leads to an active lipase biocatalyst [17]. This takes again benefit from the motility of the anchoring domains, allowing the lipase and the foldase to get into direct contact at the cell surface. In the present study we displayed human protein kinase CK2 by autodisplay on the surface of *E. coli*. Human protein kinase CK2 is an emerging target for cancer diseases [18, 19]. Despite two inhibitors of the enzymes are in clinical trials phase II at current [20, 21] more inhibitors are in need to explore the druggability and the therapeutic options of human CK2 [22]. A bottleneck in the search for new inhibitors is the availability of the target, i.e. human protein kinase CK2 for testing. Protein kinase CK2 is a heterotetramer, consisting of two subunits, a catalytic α-subunit (which can be replaced in some case by an αʹ-subunit) and a so-called regulatory β-subunit. We expressed and displayed the α- and the β-subunit of human CK2 separately on the surface of *E. coli* and showed the formation of the tetrameric holoenzyme by measuring an increased kinase activity in comparison to the α-subunit being displayed alone, by activity profiling using varying NaCl concentrations, and by co-immunoprecipitation experiments. Cells of *E. coli* displaying the holoenzyme could be used to determine the inhibition constants of two established CK2 inhibitors. Our results show for the first time, that it is possible to display a heterotretrameric human enzyme in an active form on the surface of *E. coli*, when using the AIDA-I autotransporter for autodisplay.

## Methods

### Bacterial strains and culture conditions

*Escherichia coli* BL21(DE3) [23] was used for the expression of autotransporter fusion proteins. Bacteria were routinely grown at 37°C in lysogeny broth (LB) containing 50 mg/L carbenicillin or 30 mg/L kanamycin or both, depending on the plasmid-encoded antibiotic-resistance factor(s) they carried. For expression and activity studies, LB medium was supplemented with 10 µM ethylenediaminetetraacetate (EDTA) and 10 mM 2-mercaptoethanol. Routinely, an *E. coli* liquid culture was grown overnight, and an aliquot of this culture (200 µL) was used to inoculate fresh LB medium (40 mL). Cells were cultivated at 37°C with shaking (200 rpm) until an OD_578_ of 0.6 was reached. Protein formation was induced by adding isopropyl-β-d-thiogalactopyranoside (IPTG, 1 mM final concentration) for 60 min at 30°C.

### Design of autotransporter fusion genes and expression plasmids

**Figure 1:**
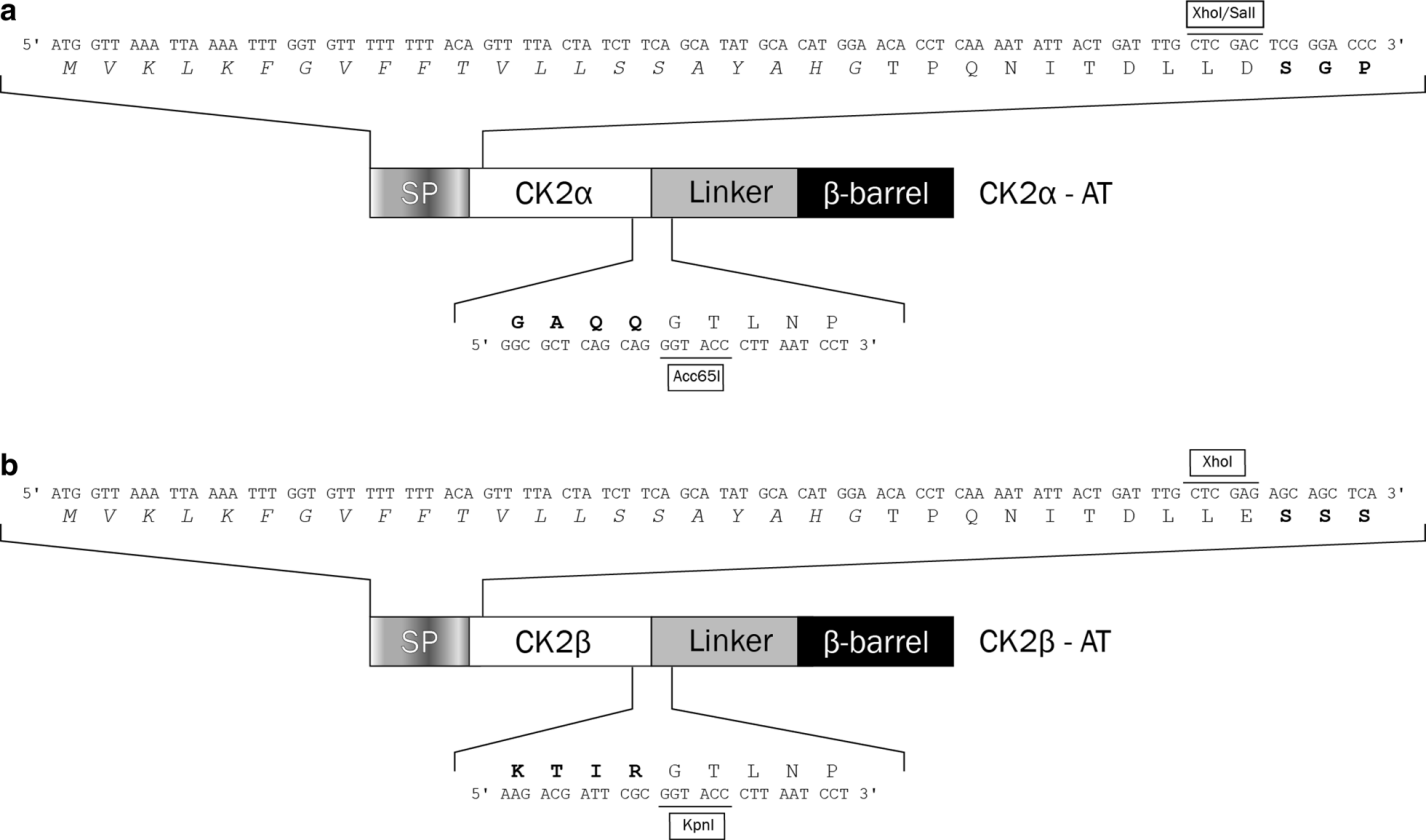
Structure of the **a** CK2α- and **b** CK2β-autotransporter fusion proteins. The restriction endonuclease recognition sites that were used for construction of the corresponding fusion genes are annotated and surrounding DNA- and amino acid sequences are given. The fusion protein domain sequence is as follows: the N-terminal signal peptide is needed for translocation through the bacterial inner membrane (*sequence in italics*) and is cut subsequently, the *open reading frames* of CK2α (**a**) or CK2β (**b**) are referred to as the passenger domain, which is supposed to be presented at the cell surface (*given in bold letters*). Linker and β-barrel domain facilitate translocation through and anchoring in the outer membrane.

In order to prepare a CK2α-coding passenger domain, human CK2α cDNA (CSNK2A1) was amplified by PCR using the two oligonucleotides Pr199 (5′-CCA GTCGAC TCG GGA CCC GTG CCA AGC AGG GCC AGA GTT TA-3′) and Pr200 (5′-AAGGTACCC TGC TGA GCG CCA GCG GCA GCT GGA ACA-3′) as primers and. The PCR product of 1,187 bp was a sequence without start- and stop codon, flanked by restriction endonuclease recognition sites (a SalI site at the 5′ end and an Acc65I site at the 3′ end). After cleavage with SalI and Acc65I, the fragment was ligated in-frame into an autodisplay plasmid (pBL002) that already contained an autodisplay fusion gene encoding signal peptide, passenger, linker and β-barrel (Figure 1). The plasmid pBL002 is a derivative of pBL001, which has been described before [17], but contained a 1.4 kBp NOX-encoding fragment in the XhoI/KpnI cloning site of pBL001. This passenger-encoding domain was removed by a restriction digest with XhoI and Acc65I, yielding a plasmid backbone with compatible ends for the CK2α insert. The resulting 6,008 bp expression plasmid pCK2α-AT carried a 2,613 bp autotransporter fusion gene for surface display of CK2α. For autodisplay of the β-subunit of CK2, a previously described autotransporter plasmid pJJ004 [14] was used as expression vector. The adrenodoxin-encoding passenger domain of pJJ004 was replaced by CK2β. Therefore, human CK2β cDNA (CSNK2B) was amplified by PCR with primers Pr201 (5′-AACTCGAGA GCA GCT CAG AGG AGG TGT C-3′) and Pr202 (5′-AAGGTACCG CGA ATC GTC TTG ACT GGG CT-3′). PCR results in CK2β coding DNA fragment devoid of start- and stop-codon but with flanking recognition sites for endonucleases XhoI (5′ end) and KpnI (3′ end). After cleavage by XhoI and KpnI, the fragment was ligated in-frame into XhoI/KpnI cleaved vector pJJ004, replacing the adrenodoxin-encoding domain and forming 7,724 bp plasmid pCK2β-AT containing the 2,082 bp autotransporter fusion gene.

### Outer membrane protein preparation and trypsin accessibility

After induction of protein formation, *E. coli* BL21(DE3) strains with either one or two plasmids, or without plasmid as controls, were harvested by centrifugation (3,850×*g*, 4°C, 10 min) and washed twice with 0.2 M Tris/HCl (pH 8.0). Outer membrane proteins were enriched according to the rapid isolation method of Handke et al. [24] with modifications as described previously [25]. In detail, cells were lysed by adding lysozyme (0.04 mg/mL end concentration) in the presence of 10 mM saccharose and 1 μM EDTA in a final volume of 1.5 mL of Tris–HCl (0.2 M, pH 8), followed by an incubation for 10 min at room temperature. Subsequently aprotinin (10 µg/mL), phenylmethylsulfonyl fluoride (PMSF) (0.5 mM), as well as 5 mL of extraction buffer (50 mM Tris–HCl pH8.0, 10 mM MgCl_2_, 2% Triton X 100) and DNAseI (10 µg/mL) were added. The samples were incubated on ice for 30 min and centrifuged for 5 min at 4°C (3,850×*g*) to remove intact bacteria and large cell debris. The supernatants representing the clarified bacterial lysate were transferred to fresh tubes and centrifuged at higher speed (38,700×*g*) for 30 min at 4.0°C to obtain the membrane protein fraction. The resulting supernatants, containing soluble cytoplasmic and periplasmic proteins, were completely aspirated. The pellets were suspended in 10 mL phosphate-buffered saline (PBS) plus 1% sarcosyl (*N*-lauryl sarcosinate, sodium salt) and centrifuged again (38,700×*g*, 60 min, 4°C). The supernatant after this step contained the sarcosyl-soluble cytoplasmic membrane proteins and was completely aspirated. The sediment representing the outer membrane protein fraction was washed twice with 10 mL of water and taken up in 30 µL purified water for SDS-PAGE. Because the outer membrane proteins with surrounding parts of the outer membrane lipids, as obtained thereby, are actually insoluble in water, this represents a suspension of outer membrane vesicles with an average diameter of 180 nm, containing the protein of interest [26]. For whole-cell protease treatment, *E. coli* cells were harvested, washed and suspended in PBS (1 mL). Trypsin (Fluka, Sigma-Aldrich, Deishofen, Germany) was added to a final concentration of 2.5 g/L and cells were incubated for 1 h at 37°C. Digestion was stopped by adding 5 mL 10% fetal calf serum (FCS) in 0.2 M Tris/HCl. After washing the cells twice with 5 mL 0.2 M Tris/HCl the outer membrane fraction was isolated. For SDS-PAGE, the outer membrane sample was dissolved in 20–40 µL PBS, depending on the yield. For co-immunoprecipitation it was dissolved in 20–40 µL kinase buffer (50 mM Tris/HCl, 100 mM NaCl, 10 mM MgCl_2_, 1 mM DTT, pH 7.5). Samples were stored at 4°C no longer than 2 days.

### SDS-PAGE and Western blot

Protein samples were diluted with SDS sample buffer (100 µM Tris/HCl, pH 6.8 with 4% SDS, 0.2% bromphenol blue and 20% glycerol). Prior to separation, samples were boiled for 20 min and loaded onto a SDS-Gel containing 7.5 or 10% acrylamide. PAGE Ruler unstained or prestained protein marker (Fermentas, St. Leon-Roth, Germany) was used as a molecular weight standard. After separation, the gel was stained with Coomassie brilliant blue G250 (Serva, Heidelberg, Germany). For Western blot, samples were separated via SDS-PAGE as described above using PAGE Ruler prestained protein marker (Fermentas, St. Leon-Roth, Germany) and were subsequently transferred onto a PVDF membrane by tank-blotting. After blocking for 1 h in TBST [TBS (pH 7.4) containing 0.1% Tween 20] supplemented with 5% dried milk, the blot was incubated for 4 h with an anti CK2β-serum (polyclonal rabbit serum, raised against amino acids 206–215 of CK2β-subunit, Serum# 32, [27]), diluted 1:5,000 in TBST, followed by three washing steps with TBST. The membrane was then incubated for 1 h with the secondary antibody [HRP-coupled anti rabbit IgG (#AO545, Sigma-Aldrich, Deishofen, Germany), dilution of 1:10,000 in TBST] followed by two washing steps with TBST and one with TBS. The immune complex was visualized via chemiluminescence (ImmunoCruz kit, Santa Cruz biotechnology, Heidelberg, Germany).

### Co-immunoprecipitation

250 µg protein A/G magnetic beads (Pierce-Thermo Scientific, Rockford, IL, USA) were mixed with 10 µg monoclonal mouse antibody #1A5, (mouse monoclonal antibody against CK2α [28]) in 500 µL binding buffer (150 mM NaCl, 25 mM Tris/HCl, 1 mM EDTA, 1% NP40, 5% glycerol, pH 7.4). After 15 h at 8°C under gently shaking, the supernatant was discarded. The antibody-loaded beads were washed and suspended in 500 µL binding buffer and mixed with 25 µL *E. coli* outer membrane fraction. The beads were precipitated after 4 h at 22°C and the supernatant was collected. The beads were washed twice with binding buffer, and once with deionized water, before the immune complex was eluted by heating (95°C for 20 min) in SDS sample buffer containing 200 mM DTT. Eluted proteins (precipitate) and supernatant were analyzed by SDS-PAGE and Western blot analysis using the polyclonal rabbit anti-CK2β-serum [27].

### Radiometric whole-cell CK2 activity

CK2-activity of whole bacterial cells was determined in a radiometric assay usually applied for in vitro testing of the purified enzyme [29]. The transfer of a [^32^P]-phosphate from ɣ[^32^P]-ATP onto the CK2-specific substrate peptide RRRDDDSDDD [30] is quantified. Here, *E. coli* cells presenting CK2 subunits were tested, instead of using the purified enzyme. *E. coli* strains were grown and protein production was induced as described above. Cells were harvested by centrifugation (3,850×*g*, 4°C, 10 min) and washed three times with kinase buffer. Cells were suspended in 20 µL kinase buffer to reach an optical density at 578 nm (OD_578_) of 2.5 (2.15 × 10^8^ cells/mL). In case of inhibitor testing, cells were suspended in 19.5 µL kinase buffer to reach an OD_578_ of 2.56. Inhibitor was dissolved in pure DMSO and 0.5 µL of inhibitor solution was mixed with the cells and incubated at 37°C for 10 min. The reaction was started by adding 30 µL hot assay buffer (150 mM NaCl, 25 mM Tris/HCl, 5 mM MgCl_2_, 0.19 mM substrate peptide RRRDDDSDDD, 0.6 µCi ɣ[^32^P]-ATP, 100 µM ATP, pH 8.5), diluting the cell suspension to reach OD_578_ = 1 in a final volume of 50 µL (equals 8.6 × 10^7^ cells/mL). CK2 activity was calculated via measuring the amount of incorporated radioactive phosphate residue in counts per minute (cpm) after binding the peptide to P81 ion exchange paper and scintillation counting. Inhibition by TBB was determined after pre-incubating CK2 with the inhibitor for 10 min before adding the hot assay buffer.

### Whole-cell CK2 activity assay and inhibition testing by capillary electrophoresis

The enzymatic activity of surface-displayed protein kinase CK2 and the IC_50_-values were determined using a recently published CE-assay [29] with slight modifications. In summary, 78 µL of the prepared bacteria suspension in kinase buffer were supplemented with 2 µL of the test-compound dissolved in DMSO and incubated for 15 min at 37°C and 400 rpm. CK2-reaction was started by the addition of 120 µL of assay buffer (25 mM Tris/HCl, pH 8.5, 150 mM NaCL, 5 mM MgCl_2_, 1 mM DTT, 167 µM substrate peptide RRRDDDSDDD and 100 µM ATP) and was carried out for 60 min at 37°C and 400 rpm. Final concentration for the substrate peptide RRRDDDSDDD was 100 µM and for ATP 60 µM with a cell concentration of OD_578_ = 2 (equals 1.72 × 10^8^ cells/mL) in a total volume of 200 µL. After the removal of CK2-presenting bacteria by centrifugation at 3,850×*g* (10 min, 4°C), the supernatant was transferred to a 96 well microplate. CK2-reaction was terminated by lowering to temperature to 4°C and complexing divalent cations with 12.5 mM EDTA before it was analyzed by capillary electrophoresis. For the determination of IC_50_ values, a sample incubated with pure DMSO served as a control for 0% inhibition, whereas a sample without the addition of ATP served as a control for 100% inhibition. The IC_50_ value of TBB was determined using nine different concentrations in appropriate intervals ranging from 100 pM to 10 µM. For silmitasertib, dose–response data using nine concentrations in appropriate intervals ranging from 10 pM to 1 µM were analyzed for IC_50_ determination.

### Influence of NaCl on the enzymatic activity of surface displayed CK2α and CK2 holoenzyme

For this purpose, enzymatic activities of whole cell either displaying CK2α or CK2α and CK2β were determined as described above for IC_50_ values with slight modifications. Briefly, 80 µL of the bacteria suspension in kinase buffer (50 mM Tris/HCl, pH 7.5, 20 mM MgCl_2_ and 1 mM DTT) with eight different concentrations of NaCl concentrations ranging from 0 to 300 mM were incubated at 30°C and 400 rpm for 15 min. CK2-reactions were initiated by the addition of 120 µL of assay buffer (25 mM Tris/HCl, pH 8.5, 20 mM MgCl_2_, 1 mM DTT, 167 µM substrate peptide RRRDDDSDDD and 1 mM ATP) having the identical concentration of NaCl as the sample with the kinase buffer they were added to. Final concentrations in a total volume of 200 µL were 100 µM for the substrate peptide and 600 µM for ATP with a final cell density of OD_578_ = 2, which corresponded to ~1.7 × 10^8^ cells/mL. For each sample enzymatic reaction was run for 60 min at 30°C and 400 rpm. CK2 displaying bacterial cells were removed by centrifugation at 3,850×*g* for 10 min at 4°C and the supernatant was transferred to a well of a microplate (96 well format). There 12.5 mM EDTA was added at 4°C to remove any free divalent cations and definitely stop the enzymatic reaction. Subsequently phosphorylation of the substrate peptide was analyzed by capillary electrophoresis as described above.

## Results

### Autodisplay of CK2 subunits α and β

For surface display of CK2α and CK2β, two artificial fusion genes were constructed, each of which contained sequences coding for essential domains for autodisplay and an open reading frame for CK2α or CK2β, respectively. The resulting fusion genes encoded a fusion protein with the following domain architecture: An N-terminal signal peptide, the passenger domain (either CK2α or CK2β subunit), a linker sequence and the β-barrel domain that anchors the surface-exposed passenger in the outer membrane of Gram-negative *E. coli*. For autodisplay of both CK2 subunits two individual fusion proteins were produced and anchored in the outer membrane: CK2α-AT containing CK2α subunit as the passenger domain (Figure 1a) and CK2β-AT, containing CK2β subunit as the passenger domain (Figure 1b). Protein production was facilitated in both cases by using plasmids with the strong T7 promoter for IPTG-inducible expression in *E. coli* via a T7 RNA polymerase gene under *UVlac5* control. *E. coli* strain BL21(DE3) was used as a host for autodisplay due to its deficiency of outer membrane protease OmpT.

The absence of OmpT avoids the cleavage of the autodisplayed protein on the cell surface. *E. coli* BL21(DE3) was transformed with either expression plasmid pCK2α (encoding CK2α-AT) or pCK2β (encoding CK2β-AT) or with both plasmids. For maintaining both plasmids in a single cell, bacteria were grown in such case with the two antibiotics carbenicillin and kanamycin. Following growth and induction of protein formation, the individual outer membrane-containing fraction of each strain was analyzed by SDS-PAGE.

**Figure 2:**
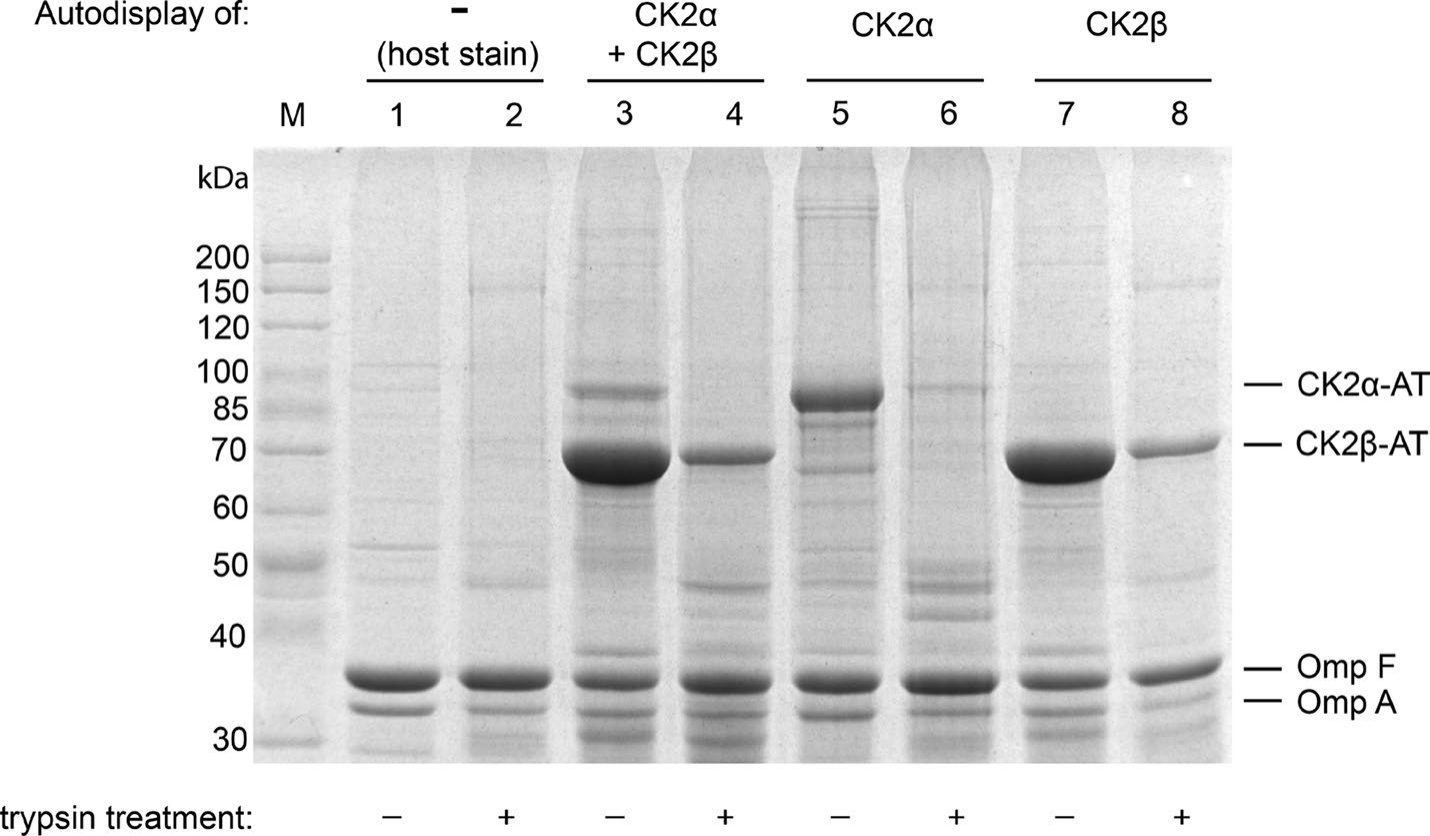
Outer membrane preparation of CK2-presenting *E. coli*. Outer membrane samples were separated by SDS-PAGE (7.5% acrylamide) and stained with Coomassie brilliant blue. For autodisplay of the individual CK2 subunits, *E. coli* BL21(DE3) pCK2α (lanes 5 and 6) or *E. coli* BL21(DE3) pCK2β (lanes 7 and 8) was used, for autodisplay of both subunits in one strain *E. coli* BL21(DE3) pCK2α pCK2β (lanes 3 and 4) was used. A fraction of each whole cell sample was treated with trypsin before preparation of outer membranes to degrade surface presented proteins facing toward the extracellular medium (lanes 2, 4, 6 and 8). The host strain without any autodisplay construct served as a control (lane 1 and 2).

In all samples, a strong expression of the autotransporter fusion proteins could be detected when comparing the samples of the plasmid containing strains with that of the host strain outer membrane fraction (Figure 2). In samples of *E. coli* BL21(DE3) pCK2α, the expected molecular weight of 95 kDa for CK2α-AT was in agreement with the appearance of a protein at an apparent molecular weight in this range (Figure 2, line 5). The same is true for expression of CK2β-AT in *E. coli* BL21(DE3) pCK2β, where the apparent molecular weight of the extra band corresponded to CK2β-AT’s predicted size of 74 kDa (Figure 2, line 7). It appeared from the density of the CK2 fusion protein bands, that expression of CK2β-AT was much more efficient than expression of CK2α-AT. This could have been due to several reasons. First of all, because both human sequences were not codon optimized for expression in *E. coli*, the presence of rare codons in CKα could interfere with its expression, whereas such effect could be less severe in CK2β. Second, it could have been the result of a gene dosage effect, namely that the copy number of the plasmid encoding Ck2α, with the pET vector backbone (ColE1 origin of replication) was less than the copy number of the plasmid encoding CK2β, based on the pCOLADuet backbone (ColA origin). Both vectors, however, were described to be medium copy number plasmids with about 15–40 copies per cell [31]. At this point, it seems unlikely, that Coomassie staining of CK2α was less efficient than staining of CK2β, because such a difference in Coommassie staining of both subunits was not observed when the recombinant human CK2 holoenzyme was analyzed by SDS-PAGE [32].

Following the successful individual expression of both CK2 subunits, *E. coli* BL21(DE3) was transformed with both plasmids, pCK2α and pCK2β to enable co-expression and autodisplay of CK2α-AT and CK2β-AT in a single strain. Analysis of outer membrane isolates indicated that while the expression level of CK2β-AT was identical to that in the strain displaying CK2β-AT alone, a further decrease in expression of CK2α-AT was observed in comparison to the expression of CK2α-AT alone (Figure 2, line 3). This could be a first indication, that indeed codon usage could account for this effect.

A presence of the fusion protein in the outer membrane fraction of *E. coli* alone does not prove surface exposure of its passenger domain. The addition of the protease trypsin to an *E. coli* suspension is a commonly used tool to examine passenger accessibility from the outside [33, 34]. Trypsin is considered too large for crossing the outer membrane. Thus, if the passenger domain is not exposed to the surface, trypsin is not able to access the fusion protein and it stays intact. In case the passenger domain is directed towards the surface, the fusion protein can be digested and its molecular weight will be reduced. On the other hand, if the integrity of the outer membrane was infringed, the protease would enter the periplasm and the fusion protein would be degraded independent from its orientation. In the latter case however, OmpA would be degraded as well due to its C terminal extension which is covalently linked to the murein layer of the cell wall. For this reason, appearance of full-length OmpA in an outer membrane fraction after trypsin treatment of whole cells is an internal control for the integrity of the outer membrane and hence a prerequisite for the validity of the experiment. In conclusion, if full-length OmpA is present and the full-length fusion protein is degraded, there is strong evidence for surface exposure of the passenger domain. Trypsin treatment of whole cells displaying either CK2α, or CK2β or both subunits resulted in a substantial decrease of full-length autodisplay fusion proteins in all cases (Figure 2, lines 4, 6 and 8). Due to the high expression level of CK2β-AT, it was not degraded completely after trypsin treatment of whole cells and a faint CK2β-AT band is still visible after SDS-PAGE (Figure 2, lane 8). In contrast, the modestly expressed CK2α-AT could be hydrolyzed almost completely by trypsin (Figure 2, lane 6). The outer membrane integrity could be ensured in all samples: the marker protein OmpA appeared in its full-length form and was not affected by trypsin treatment. These results clearly indicate the surface-exposure of both CK2 subunits, when separately expressed or when simultaneously expressed in *E. coli* BL21(DE3).

### Interaction of CK2α and CK2β on the cell surface

**Figure 3:**
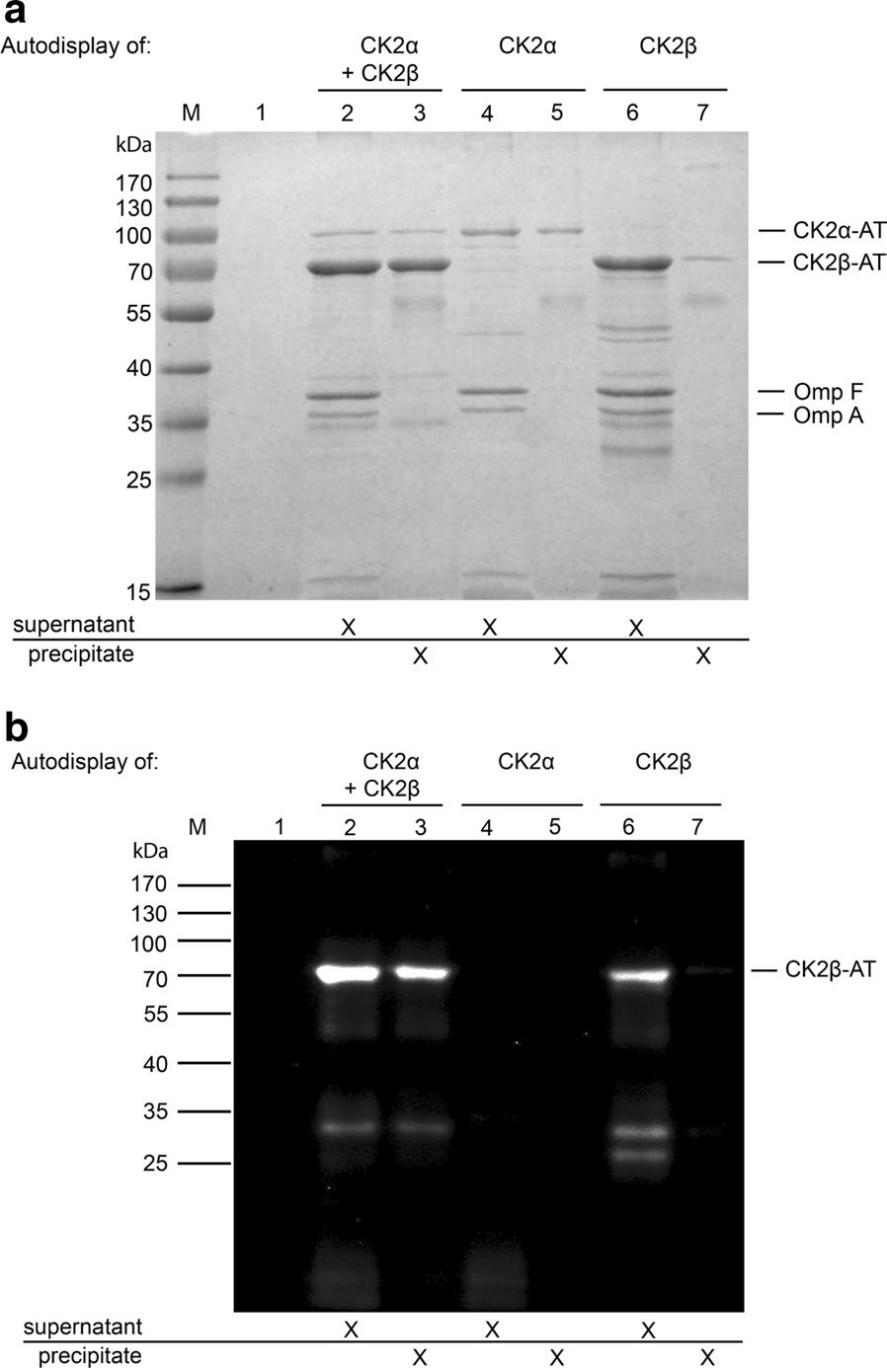
Detection of CK2 α and β subunit interaction on the bacterial surface. Outer membrane preparations of *E. coli* strains were precipitated with anti-CK2α antibody-loaded beads. The supernatant as well as the precipitate of each sample were separated on a SDS-Gel (10% acrylamide), stained with Coomassie brilliant blue (**a**) or analyzed by Western blotting using an anti-CK2-β serum for co-precipitation of CK2β-AT (**b**). OmpF and OmpA in the supernatant fraction served as an internal control for the concentration of outer membrane proteins.

The β-barrel domain anchors the autotransporter fusion protein in the outer membrane and its motility within the plane of membrane has been shown to enable passenger-driven clustering of multimeric proteins [7]. Due to the strong affinity between both CK2 subunits in vitro (K_D_ = 4 nM) [2], CK2 subunits assemble spontaneously from individually purified CK2α and CK2β. Based on this scenario, surface-presented CK2α and CK2β could interact on the cell surface to form a functional holoenzyme. The anticipated interaction of both displayed CK2 subunits was investigated by a co-immunoprecipitation approach. Therefore, outer membrane preparation of CK2 expressing *E. coli* BL21(DE3) strains were precipitated with a monoclonal anti-CK2α antibody (1A5) [28] and analyzed for co-precipitated CK2β-AT. An interaction between CK2α- and CK2β-subunit was found by the occurrence of CK2β-AT in such precipitates in SDS-PAGE and Western blot immunostained with an anti-CK2β antiserum [27] (Figure 3).

CK2α-AT precipitation was shown in all membrane samples of CK2α-AT expressing strains by the appearance of a 95 kDa protein band (Figure 3a, lanes 3, 5). Precipitation after co-expression of CK2α and CK2β yielded a clear band on a SDS-PAGE gel at the expected molecular weight of the CK2β-AT protein (74 kDa) (Figure 3a, lane 3). Moreover, in the corresponding Western blot the 74 kDa band was labeled by the anti CK2β antiserum, confirming that indeed CK2β was co-precipitated with CK2α (Figure 3b, lane 3). As can be seen in lanes 2 of Figure 3a and b, CK2β was not completely co-precipitated with the anti CK2α beads, and hence still present in the supernatant. This can be due to the much stronger expression of CK2β in comparison to CK2α on the cell surface (Figure 2, lane 3), which is supposed to result in an excess of CK2β and in consequence, in free CK2β molecules on the cell surface in addition to those interacting with CK2α.

The corresponding controls indicated that in the outer membrane fractions containing only CK2β-AT or no CK2 subunit at all, no 74 kDa protein was precipitated by the anti CK2α antibody (Figure 3a, lanes 5, 7). Application of the identical precipitation protocol to outer membrane fractions of CK2β-displaying strain resulted in a tiny amount of CK2β-AT (Figure 3a, lane 7), probably caused by an unspecific interaction of CK2β-AT with the antibody-coated beads. Due to the very small quantity of this precipitate, it does not contribute significantly to the huge amount of precipitate found in samples with CK2α-AT and CK2β-AT, and can be disregarded. In the Western blot of the controls, these findings were confirmed: just a faint band was detected when trying to precipitate the CK2β-containing sample (Figure 3b, lane 7). In samples using antibody 1A5 alone (Figure 3a, b, lanes 1), and without autodisplay of CK2β (Figure 3a, b, lane 4) no protein band of 74 kDa was detectable after co-precipitation, excluding the possibility of a mistaken identity of the CK2β-AT band. As a result, the co-precipitation of CK2β-AT and CK2α-AT showed an affinity-based complex formation of α- and β-subunit on the cell surface, which was a first indication for a possible correct folding and assembly of the CK2 holoenzyme.

### Kinase activity of *E. coli* cells displaying CK2α and CK2α + CK2β

**Figure 4:**
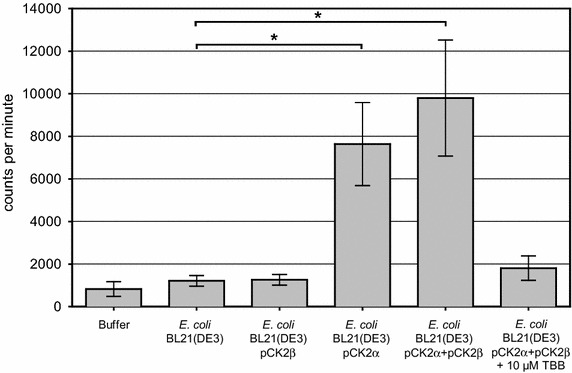
Radiometric CK2 assay using CK2-presenting *E. coli*. The amount of transferred ^32^P from γ-[^32^P]-ATP onto the synthetic substrate peptide RRRDDDSDDD was quantified in counts per minute (cpm). Reactions were catalyzed by surface display of CK2β [*E. coli* BL21(DE3) pCK2β], CK2α [*E. coli* BL21(DE3) pCK2α] and both, the α- and β-subunit of CK2 [*E. coli* BL21(DE3) pCK2α pCK2β]. Samples with the host strain or completely without cells were used as controls, having no CK2 kinase activity. *E. coli* BL21(DE3) pCK2α pCK2β was treated with the CK2 inhibitor TBB in a concentration of 10 µM prior to reaction start. The histograms display mean values (±standard deviation, n = 3, *p < 0.005).

The phosphotransferase activity of *E. coli* BL21(DE3) strains, displaying either CK2α and CK2β alone or in combination, was examined by a commonly used radiometric filter binding assay with the standard CK2-specific substrate peptide RRRDDDSDDD (Figure 4).

No kinase activity could be detected for the host strain *E. coli* BL21(DE3) (1,264 cpm) or for the strain that displayed CK2’s catalytically inactive β domain alone (1,264 cpm). In contrast, autodisplay of CK2α results in a phosphorylation of the peptide RRRDDDSDDD with a value of 7,638 cpm. The co-expression strain, presenting both CK2 subunits, yielded an even higher activity (9,802 cpm), although the expression level of the catalytic CK2α-AT was reduced by a factor of 5 compared to a separate expression (Figure 2, lanes 3, 5). If the activity is normalized to the expression level, autodisplay of both subunits yielded a >5-fold increase over the activity of the CK2α-presenting strain alone. This takes into consideration that reduction of the expression level of the catalytic subunit CKα, will lead to reduction of available active sites to the same extent. Obviously, autodisplay of CK2β along with CK2α stimulates CK2 activity, which was a further hint on a possible functional subunit interaction at the *E. coli* cell surface. In order to exclude any false-positive activity data in the whole-cell assay, the *E. coli* strain displaying both subunits was treated with the selective CK2 inhibitor 4,5,6,7-tetrabromobenzotriazole (TBB). In a concentration of 10 µM, TBB almost completely blocked CK2 activity (1,809 cpm), thus confirming that in fact the activity of surface-displayed CK2 was responsible for peptide phosphorylation. However, the high standard deviation in the radiometric whole-cell assay makes this assay format inappropriate for routine inhibitor screenings. To eliminate the unfavorable deviations, the radiometric filtration binding assay was replaced by a recently developed CK2 assay based on capillary electrophoresis (CE) [29]. Originally, this assay was described as an inhibition test for purified CK2, but the use of *E. coli* cells displaying CK2α and CK2β also resulted in a conversion of the substrate peptide RRRDDDSDDD in the CE-assay. A substrate phosphorylation of about 10–15% was achieved after 60 min by a cell suspension with an OD_578_ = 2 (1.72 × 10^8^ cells/mL) in the assay volume of 200 µL. With these settings, an initial CK2 reaction velocity (v_i_) could be recorded in all following experiments using surface-displayed CK2. In contrast to the radiometric assay, the quantification of CK2 activity resulted in a considerably lower standard deviation.

### CK2 holoenzyme formation on the cell surface

Interaction of CK2α-AT and CK2β-AT on the cell surface alone is not sufficient to prove heterotetrameric CK2 holoenzyme formation. Although CK2α/CK2β dimers have not been observed yet, and heterotetramer formation is generally considered to proceed by formation of a CK2β dimer, which subsequently collects two α-subunits to form the heterotetramer, further experimental evidence was required in order to validate that this can happen on the cell surface as well. At this point it appears worth mentioning, that the linker domain between the CK2α and β subunit passengers and the membrane anchored β-barrel domain (Figure 1a, b), comprised 160 amino acid, which were shown before to provide sufficient flexibility to allow passenger domains to interact e.g. in opposite directions without hampering enzymatic activity [9, 11]. From the number of amino acids and the flexibility of the linker domain, it was suggested, that it would not interfere with the critical N terminus of the α-subunit and in addition, the complex growth medium used to cultivate the *E. coli* cells, which contained 5 g/L yeast extract, provided sufficient zinc ions, which are required for the initial dimerization of the β-subunits.

For further experimental evidence, the NaCl dependency of surface displayed CK2α and surface co-displayed CK2α + CK2β were investigated. It has been known for a long time, that the enzymatic activity of human CK2 is dependent on the NaCl concentration in the assay. [35–38]. Interestingly, the influence of NaCl is different in case the enzymatic activity of the holoenzyme is determined, in comparison to the activity of the catalytic α-subunit alone. Whereas the α-subunit is more and more inhibited by increasing concentrations of NaCl, finally leading to an almost completely inactive enzyme, the holoenzyme is activated by increasing concentrations of NaCl [39]. Therefore we measured the enzymatic activity of cells displaying either the α-subunit alone, or the α-subunit together with the β-subunit in the CE-assay with the substrate peptide RRRDDDSDDD in the presence of different concentrations of NaCl, ranging from 0 to 300 mM.

**Figure 5:**
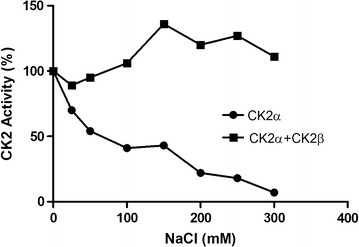
Influence of NaCl on surface displayed human CK2α (*filled circle*) and surface displayed human CK2α + CK2β (*filled square*). Increasing concentrations of NaCl ranging from 0 to 300 mM were applied, and the kinase activity of cells either displaying CK2α or CK2α + CK2β were determined by the CE assay. The substrate peptide (RRRDDDSDDD) concentration was 100 µM and the ATP-concentration was 600 µM. Activity was measured for 60 min at 30°C with cells of an OD_578_ = 2, which corresponds to 1.72 × 10^8^ cells/mL. Samples without NaCl were set to 100% activity.

As shown in Figure 5, a NaCl concentration of 25 mM strongly decreased the enzymatic activity of cells displaying only CK2α. Increasing concentrations of NaCl lead to further inhibition of Ck2α ending up with an almost negligible enzymatic activity at a NaCl concentration of 300 mM. In contrast, increasing concentrations of NaCl activated the enzymatic activity of cells displaying Ck2α together with CK2β with the highest activity obtained at a concentration of 150 mM. Further increase of NaCl to concentrations of 200 or 300 mM still activated the enzymatic activity of these cells, but not to the same extent as observed at 150 mM. The curves of activity that were obtained with the surface displayed CK2α and with surface displayed CK2α + CK2β were almost completely congruent with those obtained earlier for the free CK2α subunit and CK2 holoenzyme [39]. Consequently, it was concluded that co-expression of CK2α and CK2β on the surface of *E. coli* must have led to the formation of an active CK2 holoenzyme.

### Inhibitor testing

**Figure 6:**
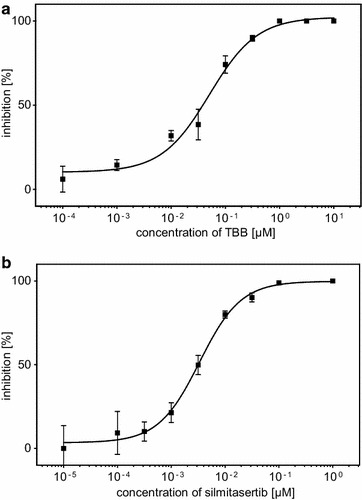
Dose-dependent inhibition of surface-displayed CK2 measured by the CE-assay. *E. coli* BL21(DE3) pCK2α pCK2β was incubated with different concentrations of **a** TBB (100 pM–10 µM) or **b** silmitasertib (10 pM–1 µM) for 10 min before the reaction was started. CK2 activity was determined by capillary electrophoretic quantification of product rate. The fractional inhibition values were calculated in respect to an uninhibited sample and are given as mean values (±standard deviation, n = 3). The IC_50_ values were obtained by fitting the dose-dependent inhibition data to a sigmoidal four-parameter logistic equation.

The easy access to the drug target CK2 by autodisplay enables the implementation of an assay without the need to purify the CK2 holoenzyme. Based on this idea, a dose-dependent inhibition of autodisplayed CK2, by TBB, a known inhibitor with a benzotriazole scaffold (4,5,6,7-tetrabromobenzotriazole) was determined in the CE assay with the substrate peptide RRRDDDSDDD. The concentrations of the inhibitor applied range from 100 pM to 10 µM and resulting enzymatic activity lead to the determination of an IC_50_ value of 0.05 µM of TBB (Figure 6a).

This IC_50_ value is in agreement with the value of 0.06 µM, obtained in a CK2-assay with purified free CK2 holoenzyme [32]. In addition, the IC_50_ value of silmitasertib (5-(3-chloro-phenylamino)benzo[c][2, 6]naphthyridine-8-carboxylic acid, CX4945), one of the most potent CK2 inhibitors known, and at present in clinical trials, was also assessed with the surface-displayed CK2 holoenzyme. The resulting IC_50_ of 3.3 nM (Figure 5b) is in line with the IC_50_ value of 3.7 nM that was published using purified CK2 holoenzyme in the CE-assay [32]. Moreover, it is in good agreement with the IC_50_ value of 1 nM that Pierre et al. [21] originally reported using a radiometric assay with a slightly different ATP concentration. The reliable quantification of in vitro inhibition and the unity of both IC_50_ values with published data obtained with the purified enzyme indicate suitability of surface-displayed CK2 for screening and characterization of CK2 inhibitors.

## Discussion

The functional autodisplay of pharmaceutically interesting target proteins on the surface of *E. coli* provides an alternative to protein purification and appears to be a powerful tool for enzyme assays. It proved especially advantageous, if the target protein is unstable in its purified form or builds inclusion bodies upon intracellular expression. The autotransporter secretion machinery, which is used by autodisplay, keeps the fusion protein in an unfolded state until it is being translocated. This feature obviously helps avoiding (intracellular) target protein inclusion bodies. Heterologous expression of human hyaluronidase 1 (Hyal-1) in *E. coli*, for example, was shown to form inclusion bodies resulting in an inactive enzyme that can exclusively be found in the insoluble *E. coli* cell fraction. Even optimized refolding yielded extremely low hyaluronidase activity [40]. The production of hyaluronidases in eukaryotic expression systems provided low yields, representing a bottleneck in early drug discovery. Autodisplay of human hyaluronidase hPH-20 circumvents the forming of inclusion bodies and results in surface-displayed, active enzyme that can be used for inhibitor screenings [41]. The concept of a quick and cost-effective surface display of target enzymes was applied to protein kinase CK2.

In this report we describe the successful surface-display of active human CK2 on the cell surface of *E. coli* by autodisplay. The fusion proteins CK2α-AT and CK2β-AT were expressed together or individually in *E. coli*, translocated to the cell surface and anchored in the outer membrane. In whole cell samples, trypsin was able to access and degrade the fusion proteins, confirming their surface exposure. Not only kinase activity of whole *E. coli* cells after autodisplay of the catalytically active subunits CK2α was detectable, moreover, combined autodisplay of CK2α and CK2β lead to the formation of an active holoenzyme at the cell surface as it was indicated by its NaCl activation. The important role of CK2β for stability and protection of CK2α against denaturing agents and proteolytic degradation was reported for the reconstituted purified recombinant holoenzyme by Meggio et al. [42]. Similarly, the activity data presented here show that CK2β-AT helps stabilizing CK2α-AT in an active conformation. A formation of CK2α/β complexes found in co-precipitation experiments mechanistically supported the possible assembly of a holoenzyme-like CK2 complex. Although passenger-driven assembly of a quaternary complex at the cell surface has been demonstrated for homomeric enzymes, autodisplay of a fully functional heteromeric enzyme has been reported in this study for the first time.

A major disadvantage in the autodisplay of CK2, which requires further improvement is the different expression levels obtained for CK2α and CK2β. This difference in expression even increased when both subunit were co-expressed on the surface of a single cell. Because the promoters used in both expression plasmids were identical, and the copy number derived from the origins of replication in both plasmids, either ColE1 or ColA were reported to be (mostly) identical (15–40 copies per cell), a different effect needs to be the reason. We analyzed the codon usage in the human sequences of CK2α and CK2β as applied here for expression in *E. coli* as fusion proteins with the autotransporter domains. The CK2β sequence contained 12 codons out of 215, which are rarely used in *E. coli*, with only three codons for tRNAs available equal or less than 3%. In contrast, the CK2α sequence contained 30 codons out of 391, which are rarely used in *E. coli*, with nine codons for tRNAs available equal or less than 3%. Moreover, six of the human codons rarely used in *E. coli* are present in CKα within a single stripe encoding amino acids residues 168–173. This could be a hint that indeed the different codon usage in both human subunits accounts for the observed differences in expression of surface displayed CK2α and CK2β. The next step will be to optimize the codon usage for both subunits with respect to the host organism *E. coli*, and find out whether this will lead to more equal expression. In addition it should lead to an increase in the activity of whole cells displaying CK2α, but of cells displaying CK2α + CK2β, as well. As indicated in the co-precipitation experiments (Figure 3a, b, lanes 2), at current, there appears to be a surplus of CK2β subunits, which are unable to gather CK2α binding partners.

We used the different NaCl dependency of CK2α and CK2 holoenzyme [39] in order to elucidate holoenzyme formation by surface displayed α- and β-subunits. The phosphotransferase activity of cells displaying CK2α was inhibited by increasing concentrations of NaCl, whereas the activity of cells displaying CKα + CK2β were activated with increasing concentrations of NaCl, as it was reported before for free CK2α and CK2 holoenzyme [39] and thus indicating the formation of functional CK2 holoenzyme on the cell surface. Moreover the curves obtained in both cases, i.e. catalytic activity versus NaCl concentration were almost identical with those obtained with the free α-subunit and the free holoenzyme, Calmodulin is a substrate protein that has been reported to be preferably phosphorylated by the α-subunit and the regulatory β-subunit in the holoenzyme almost completely inhibits its phosphorylation. This inhibitory effect may be overcome by polybasic peptides [42]. But because on one hand, this is a rather gradual effect and calmodulin is quite expensive, and on the other hand, the results obtained with the NaCl dependency of the catalytic activities were such unambiguous, we abstained from this approach. Whereas the homodimerization or homomultimerization of enzyme subunits expressed at the cell surface of *E. coli* has been reported earlier [8, 9, 16, 43, 44], this is the first report on the functional surface display of a heterotetrameric enzyme by displaying the subunits individually.

CK2 activity can easily be produced with the presented autodisplay system just-in-time for inhibition screenings or for kinetic studies of potential CK2 inhibitors. The often complex and time-consuming purification process can be avoided and “fresh” enzyme activity is available within 1 day, while storage artifacts like partially inactive or degraded enzyme species can be massively reduced. During intracellular recombinant expression and purification of CK2α for example, a fraction of the protein loses a C terminal domain of 54 amino acids due to spontaneous degradation [45]. Although the activity and the interaction of this truncated CK2α with CK2β remain unaltered, the degradation might affect some features other than kinase activity and hence should be minimized as much as possible [46, 47]. When CK2α is produced as an autodisplay-passenger, its C terminus is obviously protected from the described degradation and the full-length autodisplay fusion protein CK2α-AT was detected in SDS-PAGE. This stabilization of sensitive proteins, which require a solid support or a membrane environment for their functional integrity, is a known general benefit of autodisplay. In similar cases this was shown previously, e.g. for cytochrome P450 enzymes, which need a lipophilic surrounding for their enzymatic activity. Autodisplay of CYP106A2 and CYP3A4 yielded whole cell biocatalysts converting P450-specific substrates [10, 25].

There are a few important factors to consider when using a whole-cell biocatalyst instead of a purified enzyme. First, the applied activity assay needs to tolerate living *E. coli* organisms and the assay conditions (pH, solvents, etc.) must be chosen carefully so that the cellular integrity of *E. coli* is not disrupted. Bacterial cells remained intact during the assays as described, but in the radiometric kinase assay, the whole-cell approach may have contributed to the high standard deviation. The presence of *E. coli* in the CK2 reaction indeed showed an additional peak during CE analysis. However, the additional peak did not interfere with product quantification. Second, the assay substrate(s) and products may not be taken up actively and may not be metabolized by *E. coli*. The CK2 substrate peptide RRRDDDSDDD (including its phosphorylated form) is considered too large to cross the outer membrane of *E. coli* and in fact, we could not detect a decrease of substrate peptide concentration upon incubation with *E. coli* for >1 h. The impermeability of the cell envelope for the substrate peptide is another proof for the surface exposure of CK2, since its detected phosphorylation must be catalyzed by the surface-displayed CK2. An uptake of the CK2 co-substrate ATP cannot be fully excluded, since the outer membrane porin OmpF allows the passive diffusion of small (<600–700 Da) hydrophilic substances. In our experiments, however, surface-presented CK2 was still active >2 h after reaction start. If there is a decrease of ATP concentration caused by an uptake by the bacteria, it is in a very small order of magnitude. A recent report supports the notion that an uptake of ATP by *E. coli* even with minimum medium is very slow [48]. Third, due to the complex nature of a whole-cell biocatalyst, the concentration of active protein cannot be determined exactly. It must be deduced from SDS-PAGE of whole cell lysates or outer membrane fractions and then be compared to a stably expressed internal standard, like OmpA. At this point it needs to be taken into consideration, that the anchoring of the enzyme in the outer membrane by the β-barrel, which in consequence means, that its C terminus is covalently linked to the N terminus of the linker, which could also have an effect on enzymatic activity. This has been shown before, e.g. for surface displayed sorbit dehydrogenase, with an altered substrate specicity of the surface displayed enzyme in comparison to the free molecule [43] or for prenyltransferase, for which surface display surprisingly increased the enzymatic activity in comparison to the free enzyme [8]. For comparing the activity of surface displayed CK2α with the activity of the free enzyme, we first determined the number of CK2α molecules per single cell of *E. coli*. For this purpose, the optical densities of the bands for CK2α, as well as for OmpA, were determined in lane 5 of the Coomassie stained SDS gel shown in Figure 2. The optical density of the CK2α-autotransporter fusion protein was 2.83 times higher than that of OmpA. Because OmpA is known to have a constant number of 10^5^ molecules per single cell [49], we could calculate the molecules of the CK2α-autotransporter fusion protein per single to be 1.1 × 10^5^, by taking into consideration the different molecular weights of both proteins (95 and 35 kDa, respectively). Because the activity of a 200 µL cell suspension of an OD_578_ = 2 (equals 1.72 × 10^8^ cells/mL) without NaCl was 1.37 × 10^−5^ µmol/min, as indicated in Figure 5, the enzymatic activity of a single CK2α molecule displayed at the cell surface was estimated to be 3.66 × 10^−18^ µmol/min. The enzymatic activity of free purified CK2α was determined by CE under identical conditions with 0.2 µg of protein in 200 µL. This resulted in an activity of 3.66 × 10^−5^ µmol/min. Taking the molecular weight of free CK2α subunit into account, which is 45 kDa, an enzymatic activity of 1.16 × 10^−17^ µmol/min per single molecule could be estimated. In summary the activity of surface displayed CK2α was lowered by a factor of 3 in comparison to free CK2α and hence, the influence of the linkage to the transport unit of autodisplay had only a moderate effect on substrate conversion.

## Conclusion

Autodisplay of CK2 subunits clearly results in CK2 activity. Surface-displayed CK2α and CK2β assemble on the cell surface to an active tetrameric holoenzyme. The whole-cell CK2 autodisplay assay is suitable for inhibition studies as demonstrated with the two selective CK2 inhibitors TBB and Silmitasertib. Furthermore, it can be used to determine quantitative CK2 inhibition data such as IC_50_ values. So far we could not detect any drawbacks in replacing the purified enzyme by the surface-displayed CK2 for in vitro assays. In fact, the easy and quick availability of CK2 activity avoids a laborious purification procedure and makes the CK2 autodisplay assay a valuable tool in early drug discovery and development.
